# Repeat Prostate-specific Antigen Testing Improves Risk-based Selection of Men for Prostate Biopsy After Magnetic Resonance Imaging

**DOI:** 10.1016/j.euros.2024.05.011

**Published:** 2024-06-13

**Authors:** Petter Davik, Mattijs Elschot, Tone Frost Bathen, Helena Bertilsson

**Affiliations:** a Department of Urology, St. Olav’s Hospital, Trondheim, Norway; b Department of Clinical and Molecular Medicine, Norwegian University of Science and Technology, Trondheim, Norway; c Department of Circulation and Medical Imaging, Norwegian University of Science and Technology, Trondheim, Norway; d Department of Radiology and Nuclear Medicine, St. Olav’s Hospital, Trondheim, Norway

**Keywords:** Prostate specific antigen, Repeat testing, Magnetic resonance imaging, Targeted biopsy, Prostate cancer, Prostate adenocarcinoma, Risk model, Prediction model, Risk calculator

## Abstract

**Background and objective:**

The aim of our study was to investigate whether repeat prostate-specific antigen (PSA) testing as currently recommended improves risk stratification for men undergoing magnetic resonance imaging (MRI) and targeted biopsy for suspected prostate cancer (PCa).

**Methods:**

Consecutive men undergoing MRI and prostate biopsy who had at least two PSA tests before prostate biopsy were retrospectively registered and assigned to a development cohort (*n* = 427) or a validation (*n* = 174) cohort. Change in PSA level was assessed as a predictor of clinically significant PCa (csPCa; Gleason score ≥3 + 4, grade group ≥2) by multivariable logistic regression analysis. We developed a multivariable prediction model (MRI-RC) and a dichotomous biopsy decision strategy incorporating the PSA change. The performance of the MRI-RC model and dichotomous decision strategy was assessed in the validation cohort and compared to prediction models and decision strategies not including PSA change in terms of discriminative ability and decision curve analysis.

**Results:**

Men who had a decrease on repeat PSA testing had significantly lower risk of csPCa than men without a decrease (odds ratio [OR] 0.3, 95% confidence interval [CI] 0.16–0.54; *p* < 0.001). Men with an increased repeat PSA had a significantly higher risk of csPCa than men without an increase (OR 2.97, 95% CI 1.62–5.45; *p* < 0.001). Risk stratification using both the MRI-RC model and the dichotomous decision strategy was improved by incorporating change in PSA as a parameter.

**Conclusions and clinical implications:**

Repeat PSA testing gives predictive information regarding men undergoing MRI and targeted prostate biopsy. Inclusion of PSA change as a parameter in an MRI-RC model and a dichotomous biopsy decision strategy improves their predictive performance and clinical utility without requiring additional investigations.

**Patient summary:**

For men with a suspicion of prostate cancer, repeat PSA (prostate-specific antigen) testing after an MRI (magnetic resonance imaging) scan can help in identifying patients who can safely avoid prostate biopsy.

## Take Home Message

Repeat prostate-specific antigen (PSA) testing gives predictive information for men with suspected prostate cancer undergoing magnetic resonance imaging and targeted biopsy. Incorporation of the PSA change in risk stratification improves the predictive performance and clinical utility without adding investigations to the diagnostic pathway.

## 1 Introduction

Despite its limitations, prostate-specific antigen (PSA) remains central for detection of prostate cancer (PCa). In men with elevated PSA between 3 and 10 ng/ml and a normal digital rectal examination (DRE), the European Association of Urology (EAU) guidelines recommend repeating the PSA test before further investigations [1]. This recommendation is based on retrospective findings from the STHLM3 trial, in which 16.8% of patients with elevated PSA (3–10 ng/ml) on initial testing had a normal level of ≤3 ng/ml on repeat measurement, and on findings from the ProtecT trial, in which men with a ≥20% reduction in PSA on repeat testing had significantly lower risk of harbouring prostate cancer [2], [3]. These studies investigated repeat PSA testing in men undergoing systematic prostate biopsies triggered by a PSA threshold, without magnetic resonance imaging (MRI). Prostate MRI is currently recommended before biopsy as it contributes to avoiding unnecessary biopsy procedures and, in combination with targeted biopsies, increases detection of clinically significant PCa (csPCa) [1], [4]. Although it has been shown that repeat PSA testing provides predictive information for men undergoing systematic biopsies [2], [3], [5], whether this is the case for men undergoing contemporary diagnostic workup with MRI and targeted biopsies has not been assessed.

Risk stratification using multivariable risk calculators that incorporate MRI findings (MRI-RCs) or Prostate Imaging-Reporting and Data System (PI-RADS) scores and PSA density (PSAD) categories is currently recommended to avoid unnecessary biopsy procedures [1], [6]. However, no currently available risk stratification tools incorporate information about a change in PSA level on repeat testing [7], [8], [9], [10], [11], [12], [13], [14], [15], [16], [17], [18], [19].

The aims of this study were therefore: (1) to investigate whether repeat PSA testing gives predictive information for men undergoing MRI and subsequent targeted prostate biopsy, and (2) to develop an MRI-RC and a dichotomous biopsy decision strategy incorporating repeat PSA testing, and to assess and validate their predictive performance and clinical utility in avoiding unnecessary biopsy procedures after MRI.

## 2 Patients and methods

### 2.1 Patient population

Consecutive consenting men who underwent prostate MRI and subsequent biopsy at St. Olav’s University Hospital, Trondheim, Norway for suspected PCa because of elevated PSA or abnormal DRE were retrospectively registered. Men with PSA <100 ng/ml who had two or more PSA measurements before undergoing biopsy were included. For patients with more than two PSA results, the two latest tests preceding biopsy were used (initial and repeat PSA, respectively). Men referred from January 2019 to March 2022 were included in a development cohort, and men referred between March 2022 and May 2023 were included in a validation cohort. Patients in the development cohort were included in a previous study [19]. The study was approved by the Regional Ethical Committee of Central Norway (REC-2017/576).

### 2.2 Imaging

MRI scans were obtained using a 3-T scanner without an endorectal coil. The imaging protocol included triplane T2-weighted, axial diffusion-weighted, and axial T1-weighted sequences. Reporting was performed prospectively by expert uroradiologists using the PI-RADS scheme (version 2.0 before 2020 and version 2.1 thereafter) [6].

### 2.3 Biopsy

Patients with PI-RADS ≥3 lesion underwent targeted (± systematic) biopsies. Patients for whom a clinical suspicion of PCa persisted despite negative MRI finding (PI-RADS ≤2) underwent systematic biopsies (transrectal ultrasound [TRUS]-guided or transperineal). Sixty-nine patients with large peripheral lesions on MRI underwent TRUS biopsies, as the lesions were deemed unlikely to be missed by the treating urologist. Transrectal biopsies were performed with antibiotic prophylaxis; no antibiotic prophylaxis was used for men undergoing transperineal biopsies. All biopsies were performed under local anaesthesia. Biopsies were graded according to the International Society of Urological Pathology (ISUP) guidelines [20]. Identification of any extent of any prostate cancer was considered as PCa, and a Gleason score ≥3 + 4 (ISUP grade group ≥2) as csPCa.

### 2.4 Statistical analyses

A change in PSA level (ΔPSA) was calculated as a percentage relative to the initial PSA ([repeat PSA − initial PSA]/initial PSA × 100) and recoded into ΔPSA categories on the basis of thresholds established in previous studies [2], [3], [5]. ΔPSA categories were as follows: a PSA decrease (any, ≥5%, ≥10%, or ≥20%), stable PSA (within ±10% or within ±20%), and a PSA increase (any, ≥5%, ≥10%, or ≥20%). To assess ΔPSA as a predictor of csPCa and PCa, univariable and multivariable logistic regression models adjusted for patient age, initial PSA level, PI-RADS score, DRE findings, and prior biopsy history (biopsy-naïve vs previous negative) were analysed for the development cohort. Odds ratios (ORs) with 95% confidence intervals (CIs) are presented for each ΔPSA category. The z test was used to determine statistical significance (*p* = 0.05). We performed subgroup analyses for patients with initial PSA <10 ng/ml and patients with PI-RADS 1–3 lesions on MRI, as biopsy omission is mainly considered for these patients. Subgroup analyses were also carried out for patients with PI-RADS 3–5 lesions on MRI (MRI-positive). In addition, we assessed ΔPSA over time (PSA velocity, PSA slope, PSA doubling time, and relative ΔPSA per month [30 d]) and ΔPSA as a predictor of positive MRI findings. We developed a multivariable logistic regression model that included ΔPSA as an input variable, choosing the ΔPSA category that gave the model with the highest clinical utility at clinically relevant biopsy thresholds (5–25%). To identify the optimal way to include ΔPSA, we constructed separate multivariable logistic regression models that included each threshold for a PSA decrease (any, ≥5%, ≥10%, and ≥20%, as well as ≥30%) and a PSA increase (any, ≥5%, ≥10%, and ≥20%) as categorical input variables, as well as the percentage change in PSA as a continuous parameter. We also constructed models that included ΔPSA as a categorical variable with three categories (PSA decrease ≥10% and ≥20%; PSA stable within 10% and 20%, and PSA increase ≥10%, and ≥20%). We then performed cross-validated decision curve analysis (DCA; tenfold cross-validation with 200 repeats) of each model at the 5–25% biopsy thresholds in the development cohort [21]. The model with the highest cross-validated clinical utility in the development cohort (MRI-RC) was tested further in the validation cohort. We developed dichotomous biopsy decision rules based on PI-RADS scores and PSAD thresholds established in prior research and incorporated ΔPSA thresholds (Supplementary material) [22]. The decision rule with the highest clinical utility at the predetermined biopsy thresholds in the development cohort was tested further in the validation cohort.

The clinical utility of the final MRI-RC model and the dichotomous biopsy decision strategy was assessed via DCA in the validation cohort at predefined biopsy thresholds. We also compared these to dichotomous biopsy decision strategies based on PI-RADS scores and PSAD as suggested in the current EAU guidelines, and an MRI-RC previously published by van Leeuwen et al [1], [8]. The discriminative ability of the final MRI-RC was assessed in the validation cohort by calculating the area under the receiver operating characteristic curve (AUC). The 95% CIs for AUCs were calculated after 1000 bootstraps. AUCs were compared using the DeLong test [23]. The Mann-Whitney *U* test was used to compare continuous variables in the development cohort, and a χ^2^ test was used to compare categorical variables. Statistical analyses were performed in R (R Foundation for Statistical Computing, Vienna, Austria) [24].

## 3 Results

### 3.1 Patients

#### 3.1.1 Development cohort

We included 427 men in the development cohort; patient characteristics for the cohort are presented in Table 1. Of these men, 61% had csPCa (261/427). The increase in PSA on repeat testing was significantly greater in men with csPCa (11.2% increase, interquartile range [IQR] −1.9% to 32%) than in the group without csPCa (3.5% increase, IQR −13.1% to 18.2%; *p* < 0.001). Men with csPCa were also older, had higher PSA levels on repeat testing, higher PSAD, smaller prostates, and higher rates of suspicious DRE and of positive MRI than patients without csPCa (all *p* < 0.001). Fifteen men had a repeat PSA<3 ng/ml.

**Table 1:** Patient characteristics in the development cohort

Parameter a	Overall	No csPca b	csPCa c	*p* value d
	(*n* = 427)	(*n* = 166)	(*n* = 261)	
Age (yr)	67 (63–72)	65 (61–70)	68 (64–73)	**<0.001**
Initial PSA (ng/ml)	8.0 (5.9–12.4)	7.7 (6.0–11.0)	8.3 (5.9–15.0)	0.15
Repeat PSA (ng/ml)	8.5 (6.4–13.3)	7.6 (5.8–11.3)	9.2 (6.9–16.2)	**<0.001**
Percentage PSA change (%)	8.0 (−6.6 to 25.5)	3.5 (−13.1 to 18.2)	11.2 (−1.9 to 32)	**<0.001**
Time between PSA tests (d)	31 (20–64)	33 (22–59)	31 (19–69)	0.37
Prostate volume (ml)	48 (35–66)	59 (40–77)	42 (33–59)	**<0.001**
PSA density at referral (ng/ml^2^)	0.17 (0.11–0.27)	0.14 (0.10–0.20)	0.2 (0.13–0.32)	**<0.001**
Prior negative biopsy, *n* (%)	61 (14)	37 (61)	24 (39)	**<0.001**
PI-RADS score, *n* (%)				**<0.001**
1–2	99 (23%)	88 (89%)	11 (11%)	
3	57 (13%)	33 (58%)	24 (42%)	
4	107 (25%)	25 (23%)	82 (77%)	
5	164 (39%)	20 (12%)	144 (88%)	
Suspicious DRE, *n* (%)				**<0.001**
Yes	178 (42%)	39 (22%)	139 (53%)	
No	203 (48%)	107 (53%)	96 (37%)	
Missing	46 (11%)	20 (43%)	26 (10%)	
Biopsy method, *n* (%)				
Targeted + systematic	235 (55%)	61 (37%)	174 (67%)	
Targeted only	23 (5%)	13 (8%)	9 (3%)	
Systematic only	172 (40%)	92 (55%)	78 (30%)	

csPCa = clinically significant prostate cancer; DRE = digital rectal examination; PSA = prostate-specific antigen.

a Results for continuous variables are presented as median (interquartile range).

b None or grade group 1 prostate cancer.

c Grade group ≥2 prostate cancer.

d Mann-Whitney *U* test for continuous variables and χ^2^ test for categorical variables for csPCa versus no csPCa.

A decrease in PSA was found in 33% (143/427) of men on repeat testing: 21% (89/427) had a decrease ≥10%, and 12% (52/427) had a decrease ≥20%. Stable PSA within 20% of the initial level was found in 56% (241/427) of the patients, while 31% (134/427) had a PSA increase of ≥20%.

#### 3.1.2 Validation cohort

We included 174 men in the validation cohort; patient characteristics for the cohort are presented in Supplementary Table 1. Data for these men were not used for model development or to estimate the odds of harbouring PCa.

### 3.2 Odds of PCa for men with a change in PSA

**Fig. 1:**
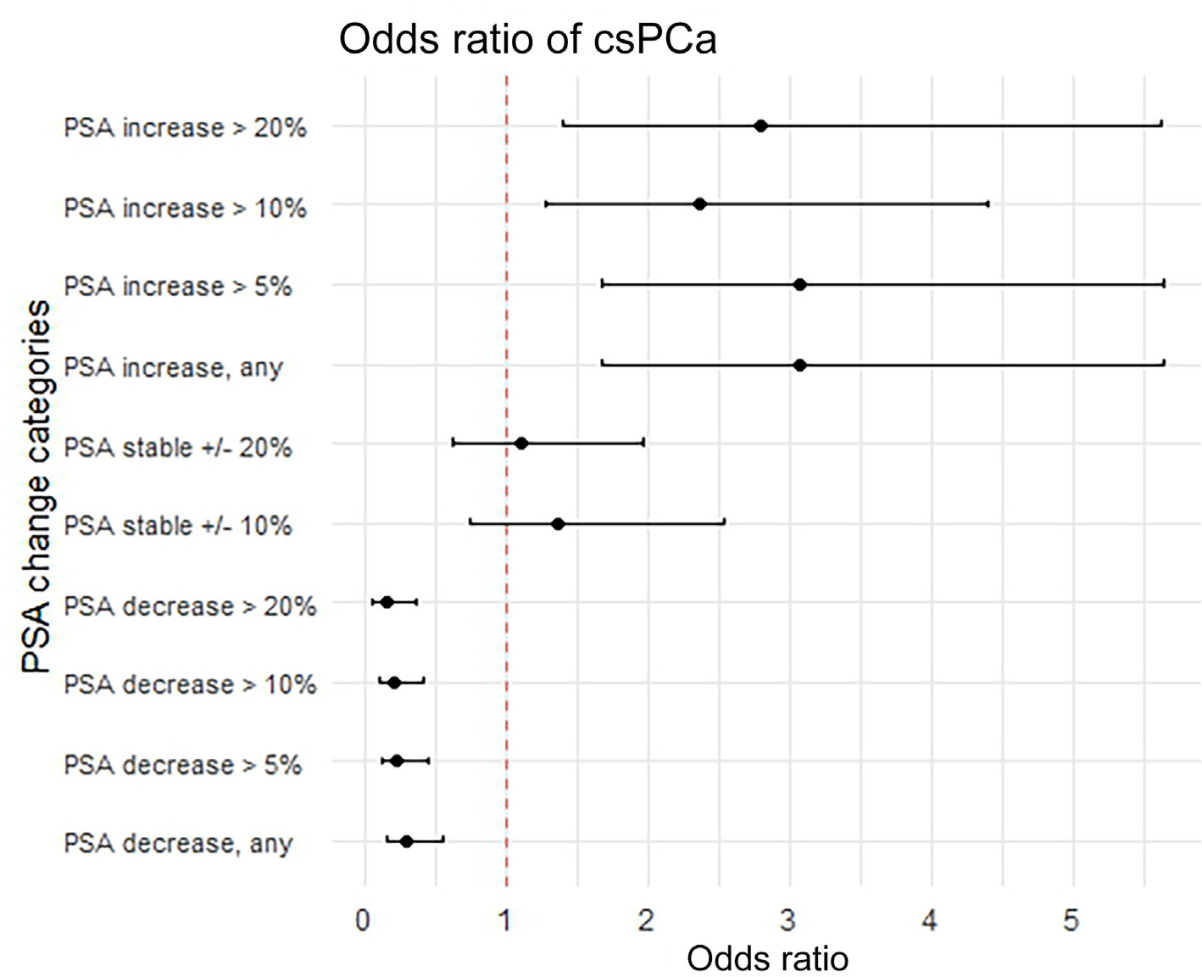
Forest plot showing the odds of clinically significant prostate cancer (csPCa) in men with versus men without a change in prostate-specific antigen (PSA) according to a multivariable logistic regression model corrected for Prostate Imaging-Reporting and Data System score, age, PSA (referral), prostate volume, prior biopsy history, and digital rectal examination applied to the development cohort (*n* = 427).

Men who had a decrease in PSA on repeat testing had significantly lower odds of csPCa and of any PCa in comparison to men without a PSA decrease (*p* < 0.001). This was the case on both univariable and multivariable logistic regression analyses adjusted for PI-RADS score, age, PSA, prostate volume, prior biopsy history, and DRE findings. Greater relative decreases in PSA were associated with lower odds of cancer, with the lowest odds of any PCa and csPCa found for men with a PSA decrease of ≥20% (Table 2 and Fig. 1). Men with an increase on repeat PSA testing had significantly higher odds of harbouring csPCa and any PCa than men who did not have a PSA increase, on both univariable and multivariable logistic regression adjusted for PI-RADS, age, PSA, prostate volume, prior biopsy history, and DRE findings. All PSA increase categories investigated were associated with higher odds of csPCa (*p* < 0.01). Stable PSA within ±10% or ±20% of the initial PSA was not significantly associated with higher or lower odds of PCa.

**Table 2:** Odds of any PCa and csPCa by PSA change in the overall cohort (*n* = 427) –

PSA change category	Any PCa (*n* = 306)		csPCa (*n* = 261)	
	OR (95% CI)	*p* value	OR (95% CI)	*p* value
PSA decrease on repeat testing				
Any PSA decrease	0.22 (0.11–0.45)	**<0.001**	0.3 (0.16–0.54)	**<0.001**
PSA decrease >5%	0.2 (0.1–0.43)	**<0.001**	0.23 (0.12–0.45)	**<0.001**
PSA decrease >10%	0.18 (0.08–0.39)	**<0.001**	0.2 (0.1–0.4)	**<0.001**
PSA decrease >20%	0.16 (0.06–0.39)	**<0.001**	0.15 (0.06–0.35)	**<0.001**
PSA stable on repeat testing				
PSA stable ±10%	1.45 (0.74–2.87)	0.28	1.37 (0.74–2.53)	0.32
PSA stable ±20%	1.47 (0.78–2.79)	0.23	1.11 (0.62–1.97)	0.72
PSA increase on repeat testing				
Any PSA increase	3.75 (1.9–7.5)	**<0.001**	2.97 (1.62–5.45)	**<0.001**
PSA increase >5%	2.95 (1.49–5.83)	**0.002**	3.12 (1.7–5.74)	**<0.001**
PSA increase >10%	2.8 (1.38–5.7)	**0.004**	2.37 (1.31–4.5)	**0.005**
PSA increase >20%	2.02 (0.96–4.3)	0.06	2.8 (1.39–5.63)	**0.004**

CI = confidence interval; csPCa = clinically significant prostate cancer (grade group ≥2); OR = odds ratio; PSA = prostate-specific antigen.

^a^Multivariable logistic regression model corrected for age, initial PSA, prostate volume, PI-RADS score, biopsy history, and digital rectal examination result.

### 3.3 Subgroup analyses

Among men with initial PSA ≤10 ng/ml (*n* = 287) those with a decrease in PSA on repeat testing had significantly lower odds of csPCa and of any PCa in comparison to men without a decrease in PSA (*p* < 0.001). Men with initial PSA ≤10 ng/ml who had a PSA increase had significantly higher risk of harbouring csPCa than those without an increase (*p* < 0.001; Supplementary Table 2). Among men with PI-RADS 1–3 findings on MRI (*n* = 156) those with a PSA decrease had significantly lower odds of csPCa than men who did not have a PSA decrease (*p* < 0.05; Supplementary Table 3). For men with PI-RADS 1–3 findings the association between a PSA increase and the odds of csPCa did not reach statistical significance (*p* = 0.053). MRI-positive patients (*n* = 328) with a PSA decrease had significantly lower odds of csPCa and of any PCa than those without a PSA decrease (*p* < 0.001; Supplementary Table 4). MRI-positive patients with a PSA increase had significantly higher odds of both csPCa and of any PCa than those without an increase (*p* < 0.05).

The PSA slope was significantly associated with csPCa (*p* < 0.001), while PSA velocity (*p* = 0.3), PSA doubling time (*p* = 0.52), and the relative PSA change per month (*p* = 0.6) were not. ΔPSA (increase, decrease, or stable) was not significantly associated with either positive (PI-RADS ≥3) or negative (PI-RADS ≤2) MRI findings on univariable or multivariable logistic regression for the overall development cohort, or for the group with initial PSA <10 ng/ml (data not shown).

### 3.4 Prediction model and decision rule incorporating ΔPSA

Inclusion of a PSA decrease ≥20% as a categorical input variable resulted in the predictive model with the highest cross-validated net benefit in the development cohort. The dichotomous biopsy decision rule that gave the highest clinical utility in the development cohort included PI-RADS scores, PSAD, and ΔPSA information, and is presented in Table 3.

**Table 3:** Biopsy decision tool based on PI-RADS score, PSA density, and PSA change

PI-RADS	Prostate-specific antigen density			
category	<0.10 ng/ml^2^	0.10–015 ng/ml^2^	0.15–0.20 ng/ml^2^	>0.20 ng/ml^2^
PI-RADS 1–2	No biopsy	Biopsy if PSA ↑ ≥20%	Biopsy if PSA ↑ ≥20%	Biopsy unless PSA ↓ ≥20%
PI-RADS 3	Biopsy if PSA ↑	Biopsy if PSA ↑	Biopsy unless PSA ↓ ≥20%	Biopsy unless PSA ↓ ≥20%
PI-RADS 4–5	Biopsy	Biopsy	Biopsy	Biopsy

↑ = increase; ↓ = decrease; PI-RADS = Prostate Imaging-Reporting and Data System; PSA = prostate-specific antigen.

#### 3.4.1 Performance for the validation cohort

When applied to the validation cohort, the multivariable model including a PSA decrease ≥20% as a categorical variable had significantly higher discrimination (AUC 0.88, 95% CI 0.83–0.93) than the model not including ΔPSA information (AUC 0.82, 95% CI 0.76–0.89; *p* = 0.01).

**Fig. 2:**
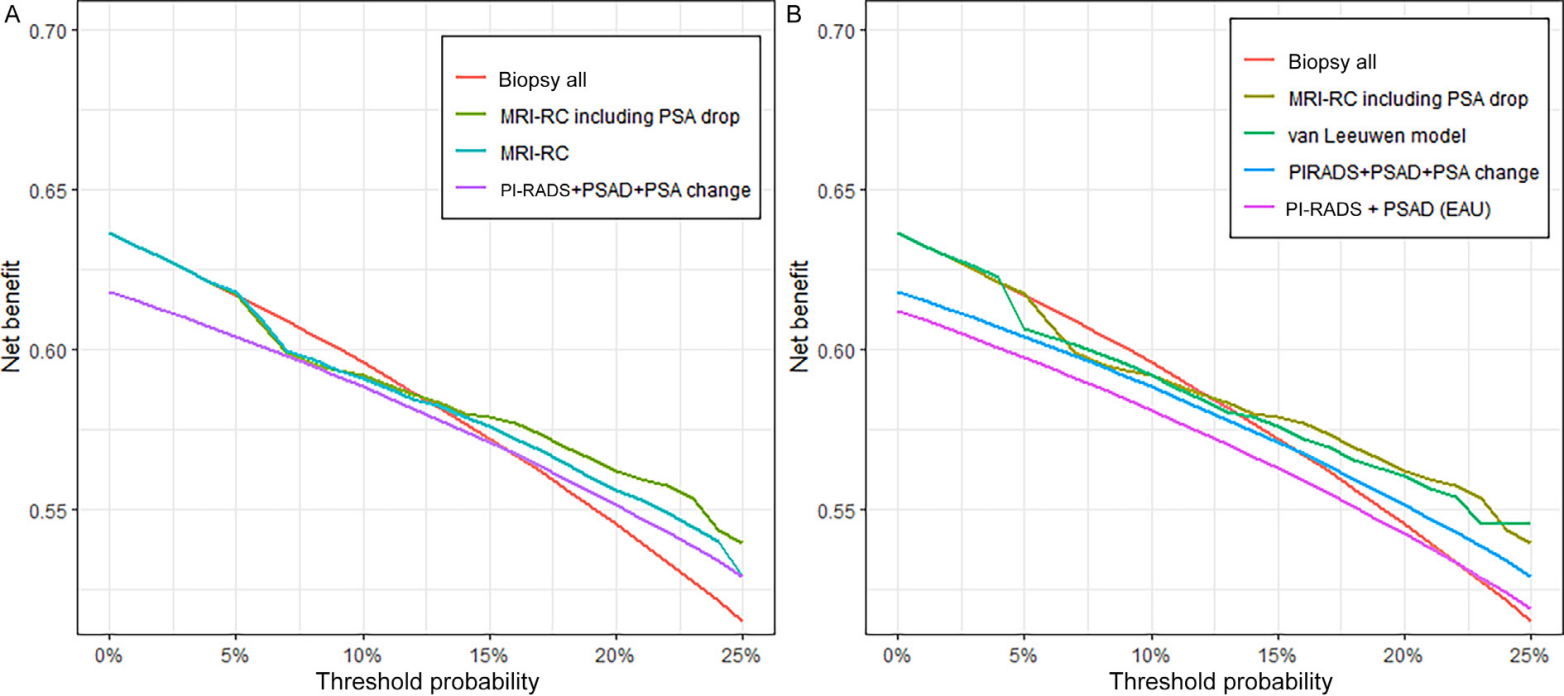
Decision curve analysis for the validation cohort (n = 174). (A) Clinical utility of the study model and dichotomous strategies. MRI-RC including PSA drop = our multivariable model including a PSA decrease ≥20%; MRI-RC = our multivariable model without information on any PSA change; PI-RADS + PSAD + PSA change = dichotomous decision rule incorporating the Prostate Imaging-Reporting and Data System score, PSA density, and PSA change (Table 3). Biopsy all = perform biopsy in all men. (B) Clinical utility of our study model and decision rule in comparison to a previously validated MRI-RC and risk stratification rule based on MRI and PSAD, as recommended in the European Association of Urology (EAU) guidelines [1]. Van Leeuwen model = MRI-RC of van Leeuwen et al [8]; PI-RADS + PSAD (EAU) = EAU guidelines decision rule [1], [25]: PI-RADS 1–2: biopsy if PSAD >0.2 ng/ml^2^; PI-RADS 3: biopsy if PSAD>0.1 ng/ml^2^; PI-RADS 4–5: perform biopsy. MRI-RC = magnetic resonance imaging risk calculator; PI-RADS = Prostate Imaging-Reporting and Data System; PSA = prostate-specific antigen;

On DCA, the multivariable model including a PSA decrease ≥20% as an input variable had higher clinical utility than the biopsy rule including information about any PSA change at all biopsy thresholds, and higher clinical utility than the multivariable model without any PSA change at thresholds >10% (Fig. 2A).

Our multivariable model and our dichotomous decision rule including ΔPSA information both outperformed risk stratification based on PI-RADS scores and PSAD suggested in the current EAU guidelines, at all biopsy thresholds [1], [25] (Fig. 2B). The multivariable model including a PSA decrease ≥20% also had higher clinical utility than the van Leeuwen model at thresholds of 5%, 15%, and 20%. A net benefit in comparison to the biopsy-all approach was observed at threshold probabilities >12.5%.

## 4 Discussion

Our study shows that repeat PSA testing gives predictive information for men undergoing MRI and targeted biopsy for suspected PCa. Men with a decrease in PSA on repeat testing had significantly lower odds of harbouring csPCa and of any PCa than men without a PSA decrease, while men with an increase on repeat PSA testing had significantly higher odds of csPCa and of any PCa.

Current guidelines recommend repeat PSA testing for men with an initial PSA between 3 and 10 ng/ml [1], but how elevated repeat PSA measurements (>3 ng/ml) should guide biopsy decisions has not been defined. In the present study we showed that incorporation of ΔPSA information improves the performance of both MRI-based multivariable prediction models and dichotomous biopsy decision strategies in predicting biopsy outcomes. Inclusion of a PSA decrease ≥20% in a multivariable MRI-RC gave the risk stratification tool with the highest clinical utility. On validation, the final MRI-RC had higher discriminative ability and clinical utility than a model without ΔPSA and than all dichotomous decision strategies assessed. It also outperformed the van Leeuwen prediction model, which has been among the best-performing models in previous studies comparing MRI-RC performance [8], [19], [26], [27].

Risk stratification to avoid biopsy procedures on the basis of PI-RADS scores and PSAD thresholds is recommended in current guidelines [1]. Because of their simplicity, this approach is preferred over multivariable MRI-RCs by some urologists, although it has been shown that MRI-RCs outperform such dichotomous decision strategies [19]. We therefore developed a dichotomous biopsy decision strategy that incorporates ΔPSA information. This novel decision strategy outperformed currently recommended risk stratification approaches based on PI-RADS scores and PSAD [1], [22], but did not outperform our MRI-RC.

As a repeat PSA test for men with suspected PCa and PSA of 3–10 ng/ml is currently recommended before further investigations, inclusion of ΔPSA information in a multivariable MRI-RC or in a dichotomous biopsy decision rule improves risk stratification of men for biopsy without necessitating additional diagnostic tests [1].

Rosario et al [2] (ProtecT) and De Nunzio et al [5] showed that a ≥20% decrease on repeat PSA testing was associated with significantly lower odds of csPCa for men undergoing PSA-triggered systematic TRUS biopsies without MRI. To the best of our knowledge, the value of repeat PSA testing for men undergoing MRI and subsequent targeted prostate biopsies has not yet been demonstrated.

PSA slope was significantly associated with detection of csPCa on biopsy, while other measures of PSA change that take the time between PSA tests into consideration were not. The final MRI-RC including a PSA decrease ≥20% as a categorical input variable had higher clinical utility than a multivariable prediction model including PSA slope (data not shown). This suggests that the relative PSA change between tests is more predictive of csPCa in comparison to PSA kinetics taking the time between samples into consideration, in keeping with findings reported by Vickers and Brewster [28].

Results were consistent across subgroups, except for men with PI-RADS 1–3 lesions on MRI. There was some evidence that the odds of csPCa were higher for men with PI-RADS 1–3 lesions and a PSA increase, but the association did not reach statistical significance (*p* = 0.053).

Only patients for whom clinical suspicion of PCa remained after MRI underwent prostate biopsy in our study, while men considered at low risk after MRI by the treating urologist were not biopsied and could therefore not be included. This reflects current recommendations regarding risk-based selection of men for biopsy and resulted in a cohort with high cancer prevalence, for which the number of men eligible for biopsy omission based on risk stratification is low and the clinical utility of risk stratification tools is expected to be limited [1]. Nevertheless, both the multivariable model and dichotomous biopsy decision tools incorporating ΔPSA were associated with a higher net benefit than the biopsy decision strategies recommended in the EAU guidelines at all biopsy thresholds, and the “biopsy all” approach at biopsy thresholds >12.5% [1].

Our study has several strengths. It is the first to investigate the role of repeat PSA testing in a contemporary diagnostic pathway in which patients undergo MRI and targeted prostate biopsies. In addition, we proposed and validated the first multivariable prediction model and the first dichotomous biopsy decision rule to incorporate information obtained from repeat PSA testing and showed that inclusion of ΔPSA information in tools supporting biopsy decisions improves their predictive performance and clinical utility.

The main limitations of our study are its retrospective design and the setting (a single, large academic centre). The study findings may therefore not be relevant to all clinical settings and should be validated in future studies on external data sets. Blood sample analyses were performed in different laboratories in our study. This can affect PSA level estimates and in turn model performance. Other limitations include the lack of a strict reference test such as template mapping biopsies and the fact that the long-term consequences of using risk stratification to avoid prostate biopsies are currently unknown.

We could not determine the optimal PSA threshold at which repeat PSA testing should be performed or the optimal time between PSA measurements in the present study, these parameters should be further investigated in future studies.

## 5 Conclusions

Repeat PSA testing gives predictive information and should be performed in men with suspected prostate cancer undergoing MRI and being considered for biopsy. Incorporation of PSA change as a parameter in MRI-based biopsy decision support tools improves selection of men for biopsy without adding investigations to the diagnostic pathway.

  ***Author contributions***: Petter Davik had full access to all the data in the study and takes responsibility for the integrity of the data and the accuracy of the data analysis.

  *Study concept and design*: Davik.

*Acquisition of data*: Davik.

*Analysis and interpretation of data*: Davik.

*Drafting of the manuscript*: Davik.

*Critical revision of the manuscript for important intellectual content*: Elschot, Bathen, Bertilsson.

*Statistical analysis*: Davik, Elschot.

*Obtaining funding*: Bathen, Bertilsson.

*Administrative, technical, or material support*: Bathen, Bertilsson.

*Supervision*: Elschot, Bathen, Bertilsson.

*Other*: None.

  ***Financial disclosures:*** Petter Davik certifies that all conflicts of interest, including specific financial interests and relationships and affiliations relevant to the subject matter or materials discussed in the manuscript (eg, employment/affiliation, grants or funding, consultancies, honoraria, stock ownership or options, expert testimony, royalties, or patents filed, received, or pending), are the following: None.

  ***Funding/Support and role of the sponsor*:** This study was supported by the Research Council of Norway (grant 295013). The sponsor played no direct role in the study.
